# Scalable and objective wound infection screening from clinical images using deep learning

**DOI:** 10.3389/fpubh.2026.1772514

**Published:** 2026-02-16

**Authors:** Chao Wang, Hongyu Wang, Jianhong Hu, Zhiyong Huang, Yan Yang, Ziming Tan, Dan Li, Li Wu

**Affiliations:** 1 Department of Burn and Plastic Surgery, West China School of Medicine, Sichuan University, Sichuan University Affiliated Chengdu Second People's Hospital, Chengdu Second People's Hospital, Chengdu, China; 2 Burns and Plastic Department, PLA No.983 Hospital, Tianjin, China; 3 State Key Laboratory of Trauma, Burns and Combined Injury, Institute of Burn Research, Southwest Hospital, Third Military Medical University (Army Medical University), Chongqing, China

**Keywords:** clinical images, deep learning, public health, Swin Transformer, wound infection detection

## Abstract

**Background:**

Wound infection is a common and clinically significant complication that can delay healing, increase healthcare costs, and contribute to inappropriate antimicrobial use. Rapid, objective, and scalable screening tools are urgently needed, particularly in resource-limited or non-specialist clinical settings. This study aimed to develop and evaluate a deep learning–based framework for automated wound infection detection using clinical wound images, with a focus on improving diagnostic consistency and supporting public health–oriented wound management.

**Methods:**

A dataset of 4,000 diverse clinical wound images was used to train and evaluate multiple deep learning models. The Swin Transformer architecture was compared with conventional convolutional neural networks. Model performance was assessed using accuracy, area under the receiver operating characteristic curve, and F1-score. To evaluate real-world applicability, model predictions were further compared with assessments made by non-specialist clinicians.

**Results:**

The Swin Transformer outperformed conventional convolutional neural networks, achieving an accuracy of 0.9025 (95% CI: 0.8695–0.9279), an area under the receiver operating characteristic curve of 0.9546, and an F1-score of 0.9042. Compared with non-specialist clinicians, the model reduced diagnostic variability and enabled earlier and more consistent recognition of wound infections.

**Conclusion:**

Deep learning applied to clinical wound images provides a scalable and objective approach for wound infection screening. Such tools have the potential to support earlier detection, reduce diagnostic variability, and improve wound management and antimicrobial stewardship, particularly in public health and resource-limited settings.

## Introduction

Wound infection is a prevalent and multifactorial clinical complication that frequently arises from trauma, surgical procedures, or chronic conditions such as diabetes and vascular diseases. These infections can delay tissue repair and significantly increase the risks of systemic sepsis, prolonged hospitalization, and mortality, highlighting the urgent need for effective wound management strategies (1–3).

Timely, accurate, and comprehensive assessment of patients and their wounds is essential to guide appropriate therapeutic interventions. Although conventional diagnostic modalities, such as microbial culture and histopathological examination, remain the gold standard, their clinical use is often limited by long turnaround times and technical constraints, which may delay urgent treatment decisions. Furthermore, positive microbial cultures can reflect mere colonization rather than true infection (3, 4). Clinicians often rely on frameworks such as the International Wound Infection Continuum (IWII-WIC) to interpret clinical signs and symptoms. However, substantial inter-observer variability between specialists and non-specialists can compromise diagnostic consistency, potentially leading to delayed recognition or mismanagement of wound infections (5). This underscores the critical need for rapid, objective, and scalable diagnostic tools that can support non-specialist clinicians, patients, and caregivers in early identification and timely referral for specialist evaluation.

Recent advances in deep learning (DL), particularly convolutional neural networks (CNNs) and Transformer-based architectures, have revolutionized medical image analysis by achieving state-of-the-art performance in image classification and pattern recognition (6). DL models trained on large, annotated medical image datasets can autonomously extract discriminative features and identify pathological patterns, providing real-time diagnostic support. Such approaches have shown transformative potential in applications including cancer screening (7–9), neurological disorder detection (10–12), hematological disorder classification (13, 14) and radiological diagnostics (15). In the specific domain of wound care, recent studies such as SwinDFU-Net have demonstrated the efficacy of hybrid architectures for identifying infections in diabetic foot ulcers (16). However, most existing models are specialized for a single wound etiology, potentially limiting their generalizability in diverse clinical environments.

In this study, we aimed to evaluate the feasibility and effectiveness of DL-based approaches for automated wound infection screening. By developing and optimizing models on a diverse clinical wound image dataset encompassing various types of wounds—including burn wounds, traumatic wounds, surgical incisions, diabetic foot ulcers, pressure injuries, and vascular ulcers—at different stages of the healing process, we sought to establish a robust, scalable, and objective pipeline. The proposed framework is designed to improve diagnostic accuracy, reduce observer-dependent variability, and streamline clinical workflows, ultimately supporting timely, precise, and equitable wound care, particularly in resource-limited or non-specialist settings.

## Methods

**Figure 1:**
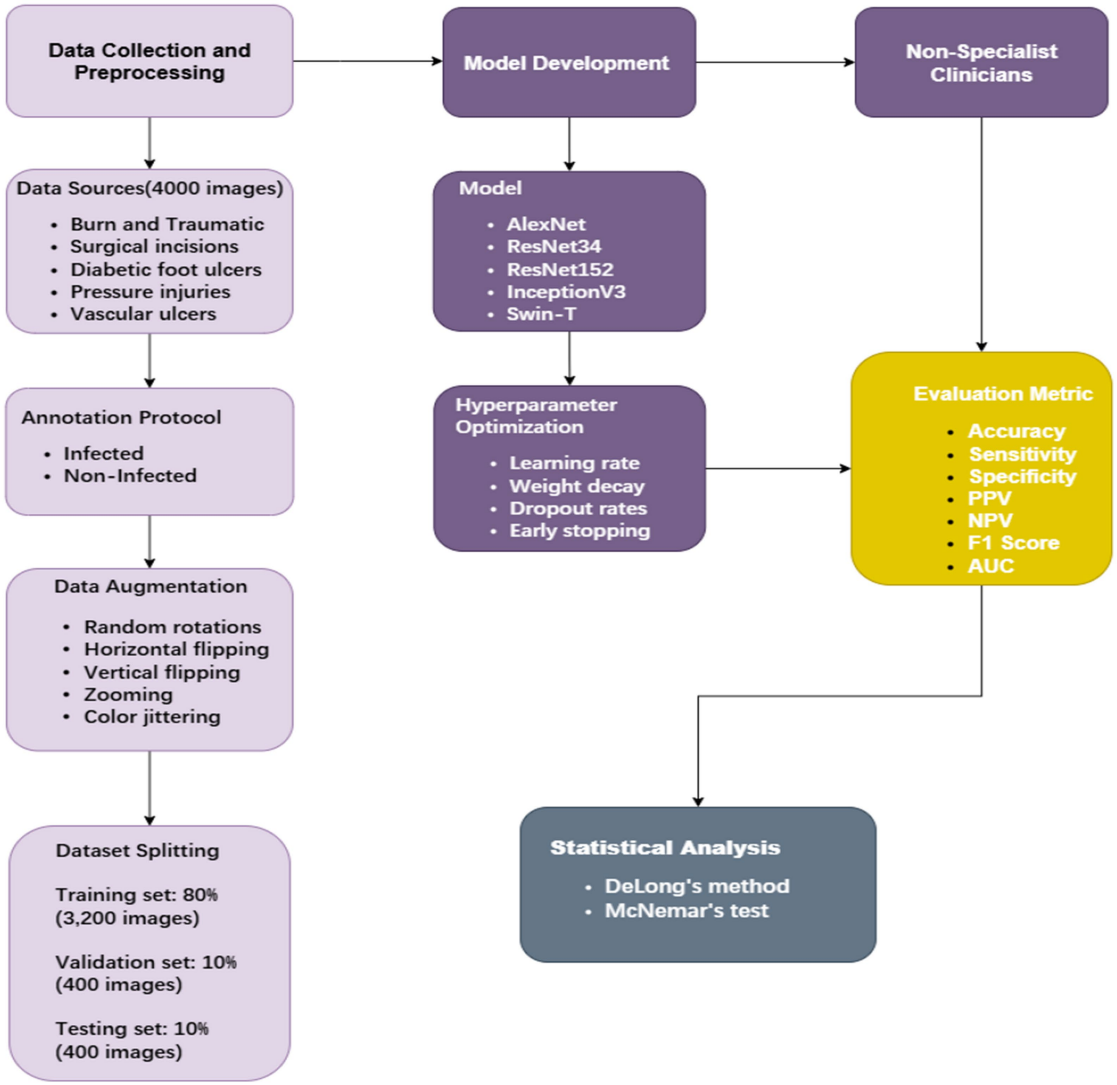
Overview of the deep learning pipeline designed to classify clinical wound images to determine whether the wound is infected.

In this study, clinical wound images were classified as infected or non-infected according to the pipeline illustrated in Figure 1.

### Data collection and preprocessing

#### Data sources

This study established a curated dataset comprising 4,000 wound images. All images were sourced from a single clinical institution (Sichuan University affiliated Chengdu Second People’s Hospital). These images encompass various types of wounds, including burn wounds, traumatic wounds, surgical incisions, diabetic foot ulcers, pressure injuries, and vascular ulcers, among other conditions, covering different stages of the healing process.

#### Data annotation

A balanced dataset of 4,000 wound images (2,000 infected and 2,000 non-infected) was utilized. To establish a high-quality ground truth, the annotation process was independently conducted by three senior wound specialists. The annotation criteria were strictly aligned with the established International Wound Infection Institute (IWII-WIC) framework (4). A multi-round cross-validation protocol was adopted. Each image was initially evaluated in a double-blind manner. Discrepancies were resolved through collective discussion until a 100% consensus was achieved. To quantify the reliability of the annotation framework, inter-observer agreement was assessed using the Fleiss’ Kappa coefficient, which yielded a value of 0.84.

#### Data augmentation

Apply data augmentation techniques such as rotation, flipping, scaling, color adjustment and Mixup to increase the diversity of the training data and enhance the robustness of the deep learning models (17, 18).

#### Data splitting

To ensure unbiased evaluation, the 4,000 images were divided into training (80%, *n* = 3,200), validation (10%, *n* = 400), and testing (10%, *n* = 400) sets using patient-level separation. This strategy ensures that all images from a single patient belong exclusively to one subset, preventing the model from learning patient-specific features. All subsets were carefully balanced to remain representative of the overall dataset and mitigate potential sampling bias.

### Model development

#### Model selection

Choose appropriate deep learning architectures for the task. AlexNet, ResNet34, ResNet152, InceptionV3, and the Swin Transformer (Swin-T) are commonly used for image classification tasks (19–24). Consider using pre-trained models (transfer learning) to leverage existing knowledge and accelerate training.

#### Model architecture

Design or modify the chosen deep learning architecture to suit the specific requirements of wound infection detection. This may involve adjusting layers, activation functions, and regularization techniques.

#### Training

Train the model using the training dataset. Implement techniques such as early stopping and learning rate scheduling to optimize performance. Use cross-entropy loss or other suitable loss functions for classification tasks.

#### Hyperparameter tuning

Optimize hyperparameters, including learning rate, batch size, and number of epochs, to achieve the best model performance. Utilize grid search or random search methods to find the optimal hyperparameters.

### Model evaluation

The performance of deep learning models is assessed using standard evaluation metrics derived from the confusion matrix. In this matrix, correct predictions are categorized as True Positives (TP) for correctly identified positive cases, and True Negatives (TN) for correctly identified negative cases. Incorrect predictions are categorized as False Positives (FP), where a negative case is incorrectly classified as positive, and False Negatives (FN), where a positive case is incorrectly classified as negative.

This study evaluates model performance using key metrics including accuracy, sensitivity, specificity, positive predictive value (PPV), negative predictive value (NPV), F1-score, and the area under the receiver operating characteristic curve (AUC-ROC). These metrics collectively help to assess the model’s effectiveness in correctly distinguishing between infected and non-infected wounds.

The evaluation metrics are defined as follows:


$$\text{Accuracy}=\frac{\mathrm{TP}+\mathrm{TN}}{\mathrm{TP}+\mathrm{TN}+\mathrm{FP}+\mathrm{FN}}$$

$$\text{Sensitivity}=\frac{\mathrm{TP}}{\mathrm{TP}+\mathrm{FN}}$$

$$\text{Specificity}=\frac{\;\mathrm{TN}}{\mathrm{TN}+\mathrm{FP}}$$

$$\mathrm{PPV}=\frac{\mathrm{TP}}{\mathrm{TP}+\mathrm{FP}}$$

$$\mathrm{NPV}=\frac{\mathrm{TN}}{\mathrm{TN}+\mathrm{FN}}$$

$$F1\;\text{Score}=\frac{2\times \mathrm{TP}}{2\mathrm{TP}+\mathrm{FP}+\mathrm{FN}}$$

AUC-ROC: Area under the receiver operating characteristic curve, computed using the trapezoidal rule (25).

All metrics were reported with 95% confidence intervals (CI) calculated via 1,000 bootstrap resamples to account for sampling variability.

Validation: Assess the final model on the test dataset to measure its performance on unseen data. Analyze results to determine the model’s effectiveness in real-world scenarios.

### Comparison with non-specialist clinicians

To assess clinical utility, we compared the diagnostic performance of the best-performing model with that of four non-specialist physicians. Each physician independently classified the same 400-image test set without access to clinical metadata or contextual information. The performance of each clinician was evaluated using the same metrics as those used for the deep learning models. McNemar’s test was used to statistically compare the misclassification rates between the best model and each non-specialist.

### Statistical analysis

Model comparison: Pairwise differences in AUC-ROC were tested using DeLong’s method for correlated ROC curves. Significance threshold: *α* = 0.05 (two-tailed).

Hypothesis: H0: AUCa = AUCBb vs. H1: AUCa = AUCb.

McNemar’s test was applied to compare error rates (FP + FN) between models on the same test set:


$$\chi^{2}=\frac{F_{\mathrm{na}}+F_{\mathrm{pb}}}{{\left(|F_{\mathrm{na}}-F_{\mathrm{pb}}|-1\right)}^{2}}$$

where *F*_na_ = false negatives of Model a, *F*_pb_ = false positives of Model b. All statistical analyses were performed using Python 3.10.5.

## Results

### Training process analysis

**Figure 2:**
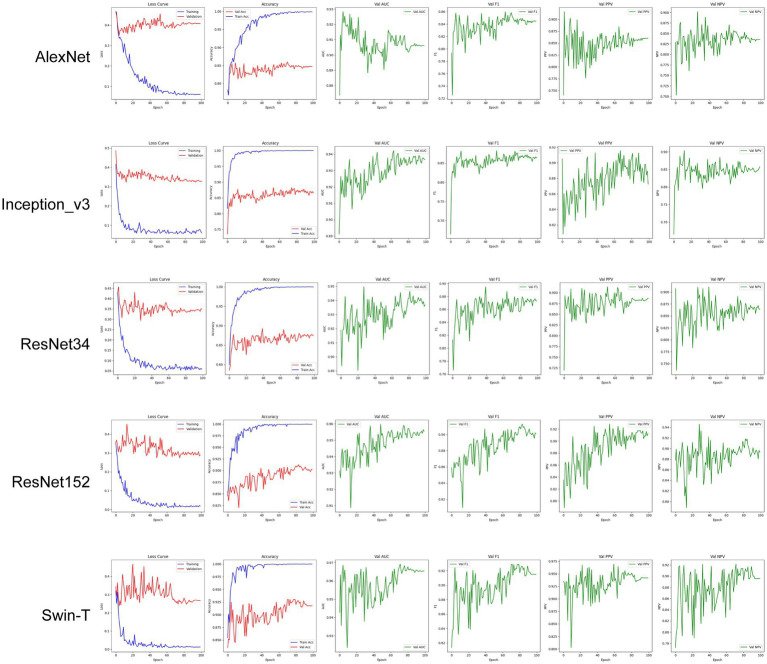
Training dynamics and validation metrics of five deep learning models.

During model training, we conducted a comparative evaluation of several widely used deep learning architectures, including Swin-T, ViT_b_16, ResNet152, ResNet34, Inception_v3, and AlexNet, to analyze their performance in automated wound infection classification. As illustrated in Figure 2, the models were trained over 100 epochs, during which multiple performance metrics were recorded, including accuracy, sensitivity, specificity, precision, F1-score, and AUC. To prevent overfitting and ensure the retention of the best-performing model, the training process incorporated early stopping and model checkpointing strategies.

### Model performance

**Figure 3:**
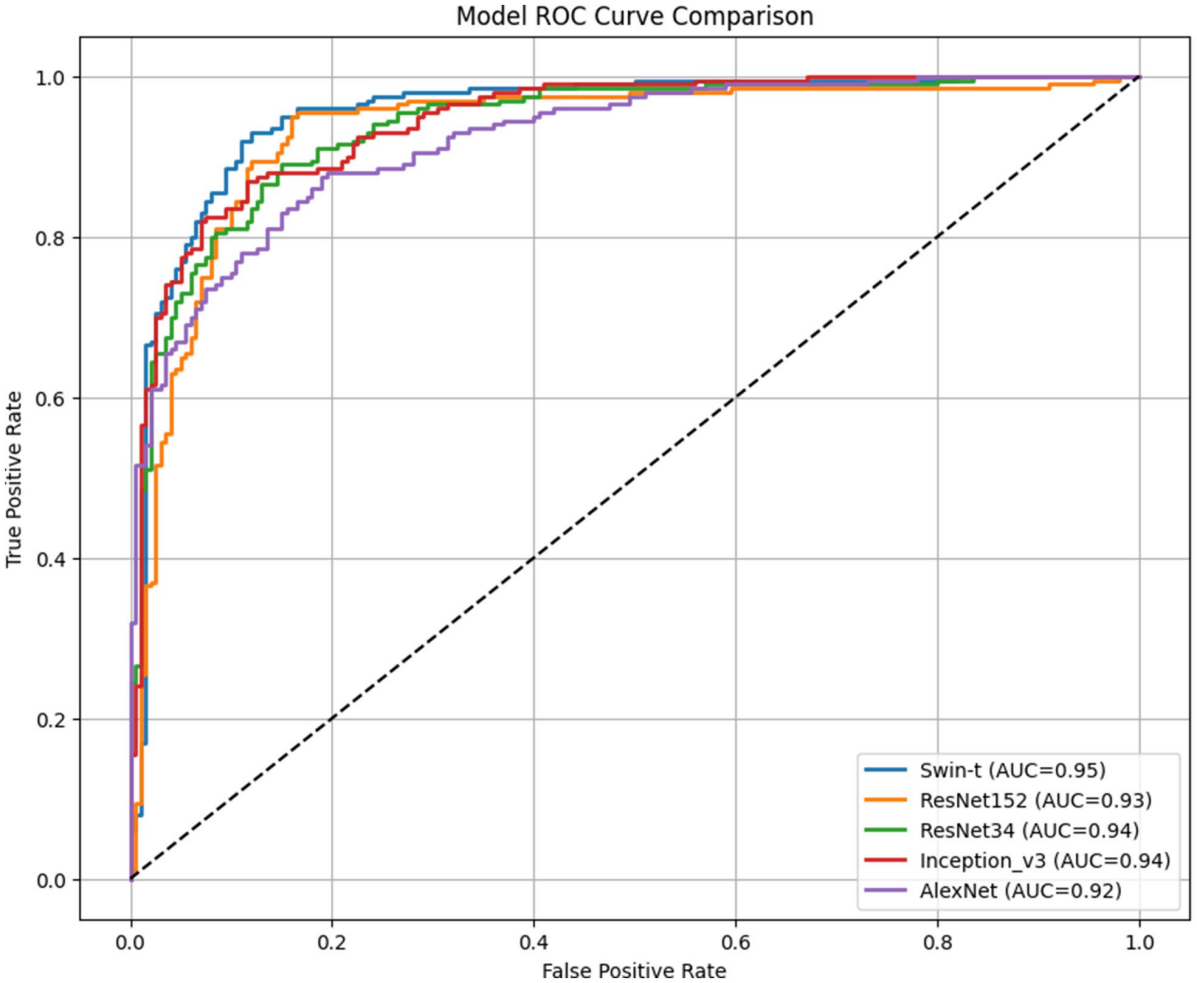
Receiver operating characteristic (ROC) curves for model comparison.

Five deep learning models were successfully trained and evaluated on the curated wound infection dataset. All models achieve AUC values exceeding 0.9 (0.92–0.95), far exceeding random classifiers (AUC = 0.5), indicating exceptional classification discrimination capabilities across all five models (Figure 3). However, the Swin-T model demonstrated the best overall performance. Specifically, Swin-T achieved an accuracy of 0.9025, AUC-ROC of 0.9546, and F1-score of 0.9042, outperforming traditional CNN architectures including AlexNet, ResNet34, ResNet152, and Inception_v3 across most evaluation metrics (Table 1). After clinical decision threshold optimization via the Youden Index, Swin-T achieved a peak accuracy of 90.50% and an AUC-ROC of 0.9546, maintaining an optimized balance between sensitivity (92.00%) and specificity (89.00%). While the ResNet152 model exhibited a higher raw sensitivity of 95.00% under its optimized threshold (0.1554), this came at the cost of a significantly lower specificity (84.00%) (Table 2). In contrast, Swin-T consistently outperformed ResNet152 in terms of F1-score (0.9064) and overall diagnostic stability, providing a more reliable decision boundary for clinical infection screening.

**Table 1:** The performance comparison of various deep learning models evaluated on the clinical wound infection dataset.

Metric	AlexNet	InceptionV3	ResNet34	ResNet152	Swin-T
Params (M)	11.97	21.79	21.29	58.15	**86.75**
NVIDIA T4(ms)	1.8 ± 0.8	17.9 ± 3.0	7.48 ± 1.7	21.8 ± 3.5	**27.7 ± 2.0**
CPU (ms)	109.3 ± 4.3	313.7 ± 7.4	80.66 ± 7.5	320.6 ± 5.7	**486.4 ± 5.2**
Accuracy	0.8325 [0.7928–0.8659]	0.8625 [0.8253–0.8928]	0.8650 [0.8280–0.8950]	0.8850 [0.8500–0.9127]	**0.9025** **[0.8695–0.9279]**
Sensitivity	0.8550 [0.7995–0.8971]	0.8450 [0.7884–0.8886]	0.8400 [0.7829–0.8843]	0.8750 [0.8220–0.9139]	**0.8850 [0.8334–0.9221]**
Specificity	0.8100 [0.7500–0.8583]	0.8800 [0.8277–0.9180]	0.8900 [0.8391–0.9262]	0.8950 [0.8448–0.9303]	**0.9200 [0.8740–0.9502]**
PPV	0.8182 [0.7603–0.8646]	0.8756 [0.8216–0.9150]	0.8842 [0.8309–0.9223]	0.8929 [0.8418–0.9288]	**0.9171 [0.8696–0.9483]**
NPV	0.8482 [0.7904–0.8922]	0.8502 [0.7953–0.8924]	0.8476 [0.7928–0.8899]	0.8775 [0.8254–0.9156]	**0.8889 [0.8388–0.9248]**
F1 Score	0.8286 [0.7863–0.8670]	0.8649 [0.8241–0.8964]	0.8683 [0.8338–0.9016]	0.8861 [0.8513–0.9169]	**0.9042 [0.8727–0.9327]**
AUC	0.9212 [0.8958–0.9424]	0.9443 [0.9221–0.9643]	0.9388 [0.9153–0.9605]	0.9320 [0.9027–0.9560]	**0.9546 [0.9324–0.9755]**

The values in brackets represent the 95% confidence intervals. Bold values indicate the performance results of the Swin-T model for comparison with other models.

**Table 2:** Comparison of model performance using default (0.5) and optimized probability thresholds.

Model	Threshold type	Optimal threshold	Accuracy (%)	Sensitivity (%)	Specificity (%)	F1-Score	AUC (95% CI)
Swin-T	**Default**	**0.5**	**90.25**	**92**	**88.5**	**0.9042**	**0.9546 (0.93–0.97)**
	**Optimized**	**0.5848**	**90.5**	**92**	**0.89**	**0.9064**	**0.9546 (0.93–0.97)**
ResNet152	Default	0.5	88.5	89.5	87.5	0.8861	0.9320 (0.90–0.95)
	Optimized	0.1554	89.5	95	0.84	0.9005	0.9320 (0.90–0.95)
ResNet34	Default	0.5	86.5	89	84	0.8683	0.9388 (0.91–0.95)
	Optimized	0.5541	87	89	0.85	0.8725	0.9388 (0.91–0.95)
Inception_v3	Default	0.5	86.25	88	84.5	0.8649	0.9443 (0.92–0.96)
	Optimized	0.5817	87.75	87	0.885	0.8766	0.9443 (0.92–0.96)
AlexNet	Default	0.5	83.25	81	85.5	0.8286	0.9212 (0.89–0.94)
	Optimized	0.4216	84.25	87.5	0.81	0.8475	0.9213 (0.89–0.94)

Optimized thresholds were determined using the Youden Index (J = Sensitivity + Specificity – 1) to achieve the optimal balance between sensitivity and specificity. Bold values indicate the performance results of the Swin-T model for comparison with other models.

**Figure 4:**
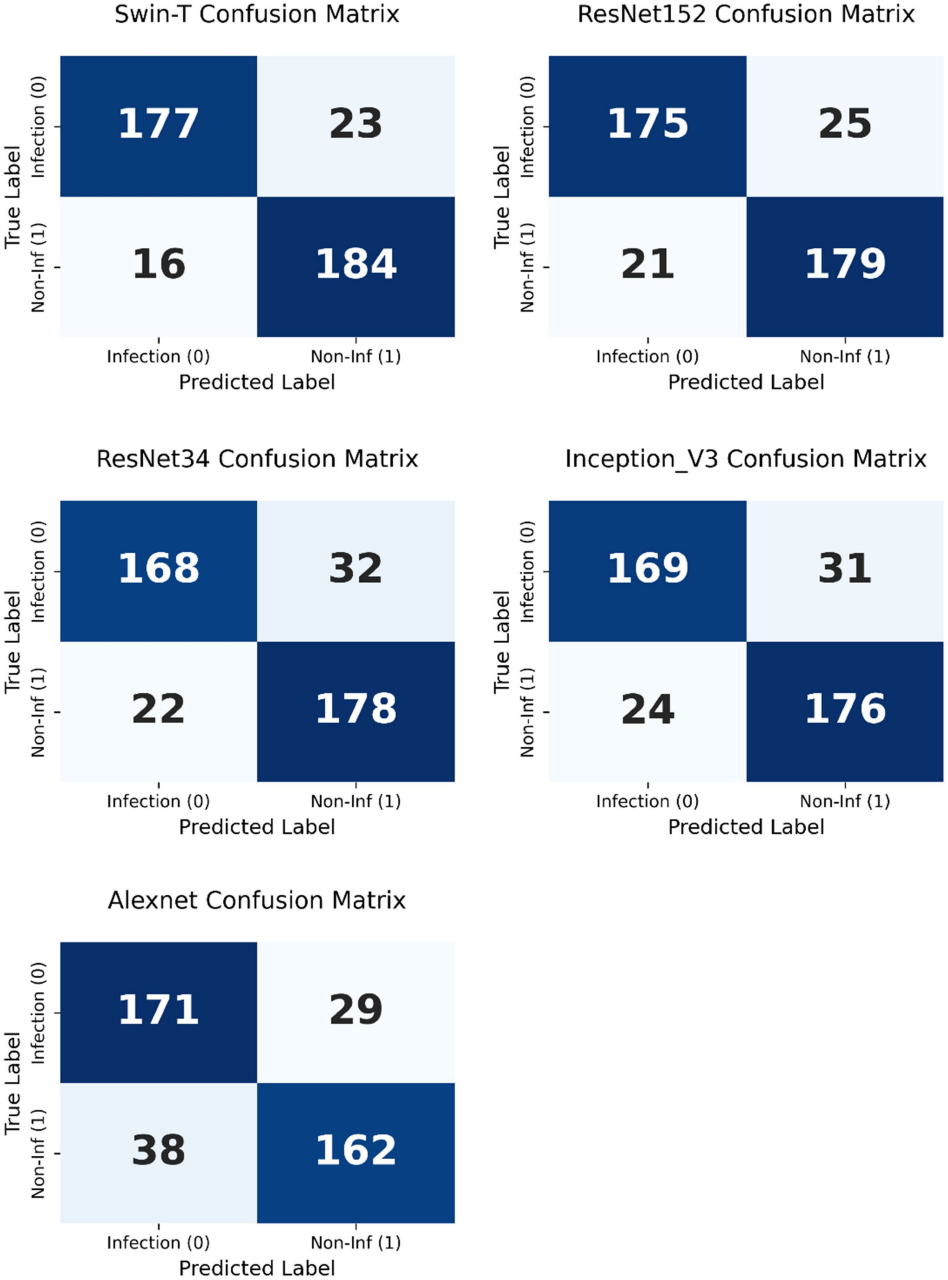
Confusion matrices of five deep learning models—AlexNet, ResNet34, InceptionV3, ResNet152, and Swin-T—used for the classification of infected versus non-infected wounds.

**Figure 5:**
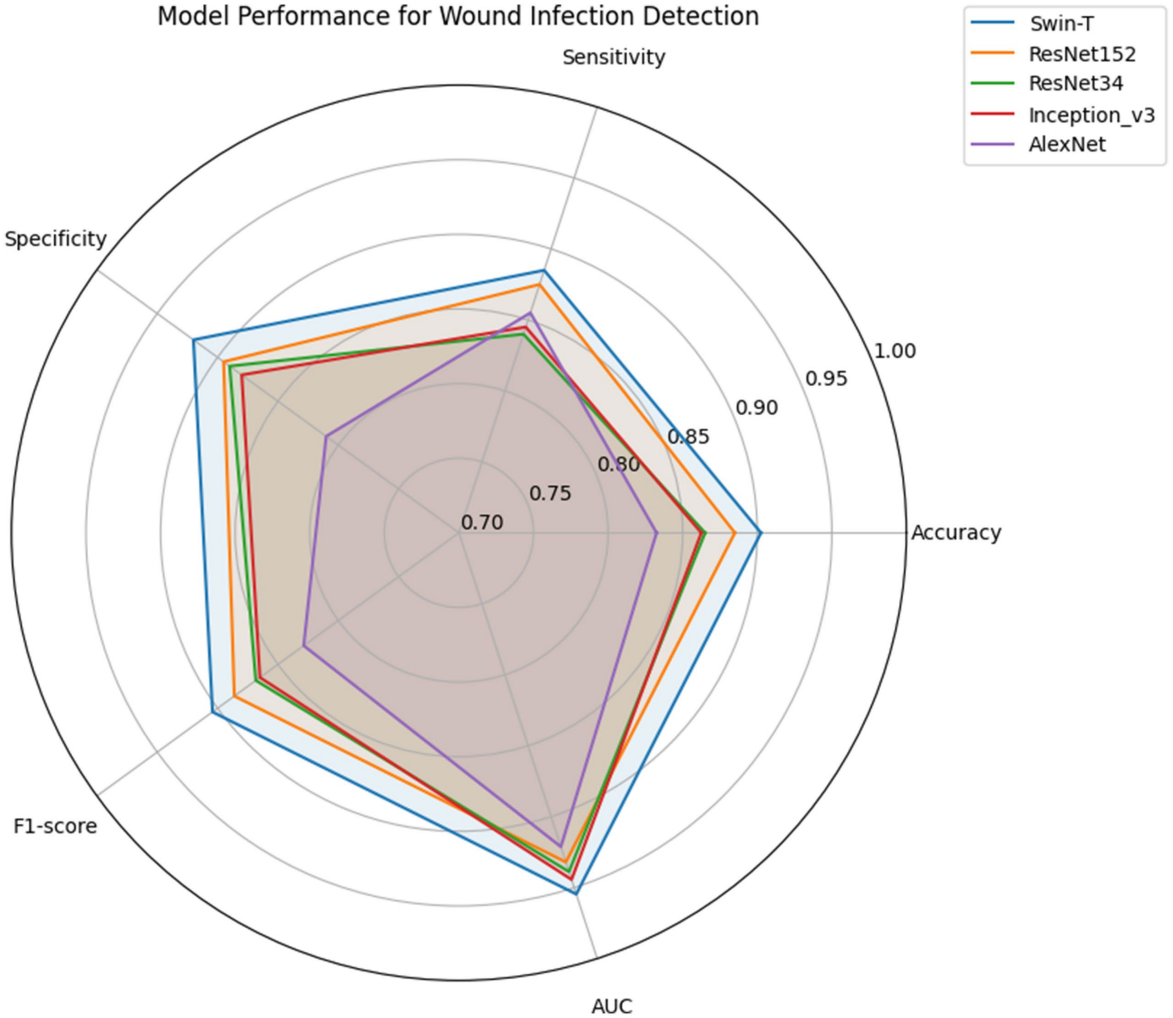
Comparative performance of deep learning models in wound infection detection. Radar plot illustrating the performance of five deep learning architectures—AlexNet, ResNet34, InceptionV3, ResNet152, and Swin-T—across five evaluation metrics: Accuracy, Sensitivity, Specificity, F1-score, and AUC.

To further interpret the diagnostic performance of each model, confusion matrices were generated based on the test set predictions (Figure 4). For the best-performing model, Swin-T, the confusion matrix revealed a high number of true positives (TP = 177) and true negatives (TN = 184), indicating strong capability in accurately detecting both infected and non-infected wounds. The number of false positives (FP = 16) and false negatives (FN = 23) remained relatively low, which supports the high specificity (0.920) and sensitivity (0.885) observed in the metric evaluations (Figure 5).

The computational efficiency of the models was evaluated across different hardware platforms, as summarized in Table 1. Among the tested architectures, the Swin-T model (approx. 86.75 M parameters) achieved a high-performance inference speed of 27.7 ± 2.0 ms on an industry-standard NVIDIA T4 GPU, significantly meeting the requirements for real-time clinical applications. Even when deployed on a standard CPU, the Swin-T model maintained a responsive inference time of 486.4 ± 5.2 ms. While lightweight models like AlexNet showed faster raw speeds, Swin-T provided a more robust balance between architectural capacity and operational latency, ensuring its feasibility for deployment in diverse clinical environments.

### Statistical significance testing

To rigorously assess performance differences, DeLong’s test and McNemar’s test were conducted between Swin-T and all other models. Statistically significant improvements (*p* < 0.05) were observed between Swin-T and AlexNet for both AUC-ROC (*p* = 0.0178) and misclassification rate (McNemar’s *p* = 0.0013). Although differences in AUC between Swin-T and ResNet34, InceptionV3, and ResNet152 did not reach statistical significance, McNemar’s test indicated Swin-T produced significantly fewer classification errors compared to ResNet34 (*p* = 0.0328) and InceptionV3 (*p* = 0.0304), suggesting a consistent advantage in practical diagnostic performance (Table 3).

**Table 3:** Statistical significance tests comparing Swin-T with other models.

Comparison	ΔAUC	DeLong p	DeLong Sig.	McNemar *χ*^2^	McNemar p	McNemar Sig.
AlexNet	−0.0334	**0.0178**	✔	10.41	**0.0013**	✔
ResNet34	−0.0158	0.1767	–	4.56	**0.0328**	✔
InceptionV3	−0.0103	0.3679	–	4.69	**0.0304**	✔
ResNet152	−0.0226	0.0993	–	0.80	0.3711	–

Bold values indicate a statistically significant difference (*P* < 0.05) when compared with the Swin-T model.

### Comparison with non-specialist clinicians

**Figure 6:**
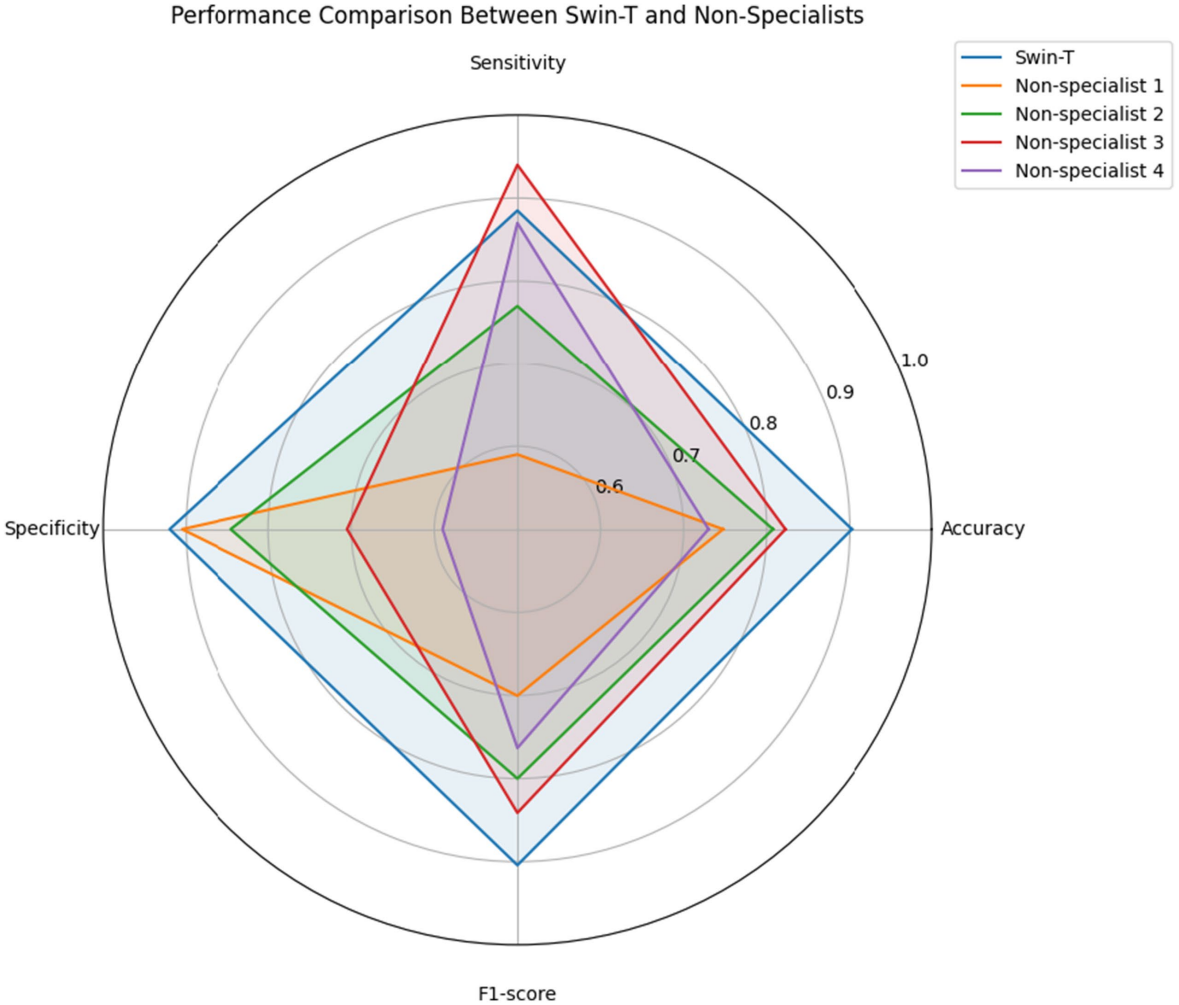
Performance comparison between the Swin-T model and non-specialist clinicians in wound infection detection. Radar chart illustrating the performance metrics—accuracy, sensitivity, specificity, and F1-score.

The Swin-T model’s diagnostic capability was further benchmarked against four non-specialist clinicians. Swin-T consistently outperformed all four clinicians in accuracy (90.25% vs. 73.00–82.25%) and F1-score (0.9042 vs. 0.7003–0.8412) (Table 4 and Figure 6).

**Table 4:** Performance comparison between Swin-T and non-specialists.

Metric	Swin-T	Non-specialist 1	Non-specialist 2	Non-specialist 3	Non-specialist 4
Accuracy	0.9025	0.7475	0.8075	0.8225	0.7300
Sensitivity	0.885	0.590	0.770	0.940	0.870
Specificity	0.920	0.905	0.845	0.705	0.590
PPV	0.9171	0.8613	0.8324	0.7611	0.6797
NPV	0.8889	0.6882	0.7860	0.9216	0.8194
F1-score	0.9008	0.7003	0.8000	0.8412	0.7632
McNemar *p*-value	**––**	**<0.0001**	**<0.0001**	**0.0010**	**<0.0001**

Bold values indicate a statistically significant difference (*P* < 0.05) when compared with the Swin-T model.

**Figure 7:**
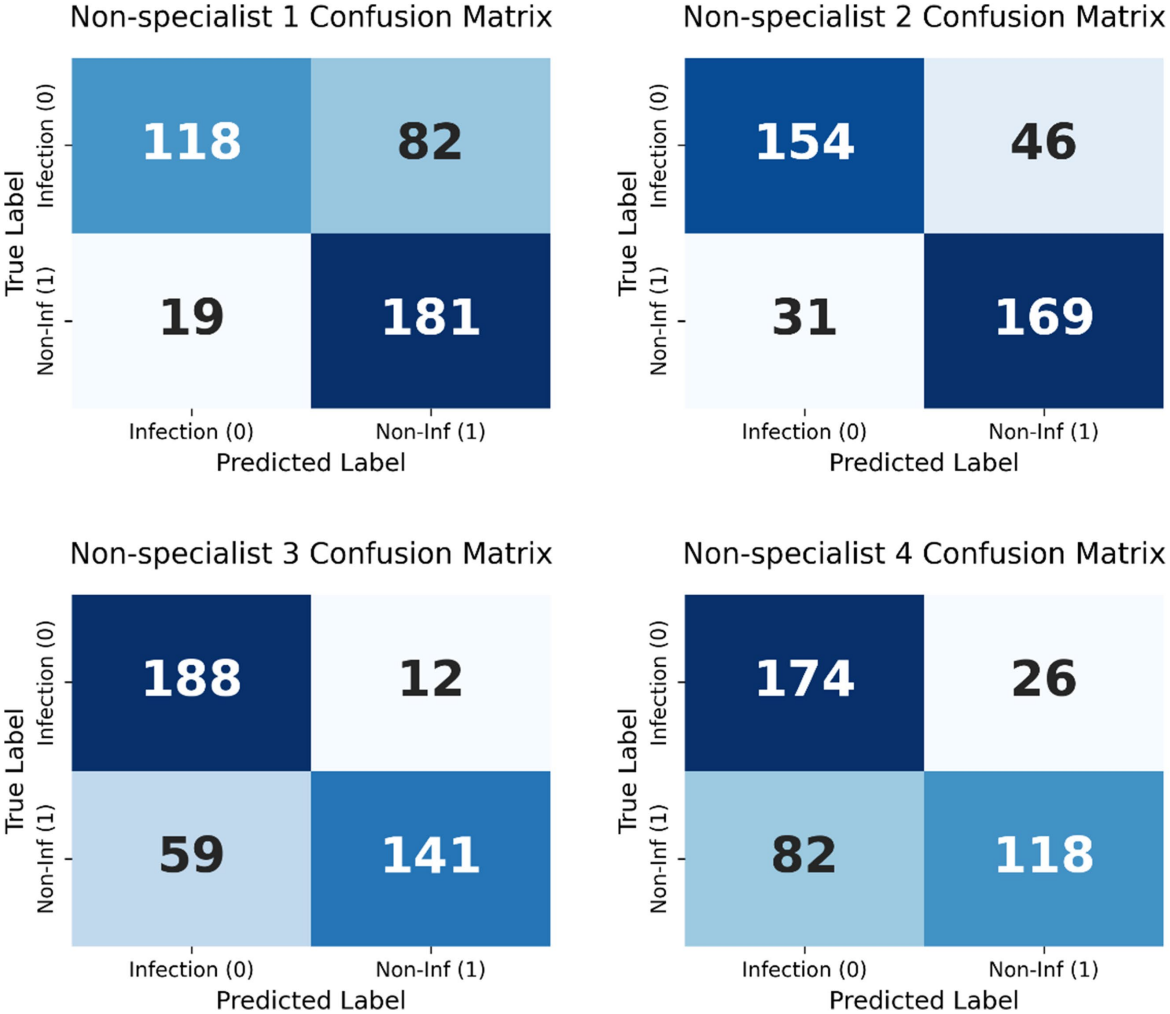
Confusion matrices of non-specialist clinicians—used for the classification of infected versus non-infected wounds.

To enable a direct comparison with the Swin-T model, confusion matrices were generated for each non-specialist clinician using the same test set predictions (Figure 7). Compared with Swin-T, confusion matrices of non-specialist clinicians revealed notable performance trade-offs. For example, Non-specialist 3 exhibited the highest sensitivity (94.0%) but also had a markedly higher number of false positives (FP = 82), leading to decreased specificity and increased unnecessary alarms. Non-specialist 1 and 4, while more conservative, missed more actual infections (higher FN), resulting in lower sensitivity. These patterns suggest a common challenge among non-specialist clinician in maintaining a balanced classification threshold, often favoring either sensitivity or specificity but not both. McNemar’s test revealed statistically significant differences (*p* < 0.01) between Swin-T and all clinicians, confirming its superior diagnostic precision (Table 4).

These results demonstrate the potential of Transformer-based deep learning models as reliable, expert-level diagnostic tools for wound infection detection. The Swin-T model achieved high accuracy across diverse wound types, suggesting clinical viability as an assistive tool for early infection detection, particularly in resource-limited or primary care settings.

## Discussion

This study proposes a robust and clinically relevant deep learning framework for the automatic detection of wound infections, in which the Swin-T model outperforms traditional CNN architectures. Swin-T demonstrated superior performance in terms of accuracy (90.25%), F1 score (0.9042), and AUC-ROC (0.9546), indicating that Transformer-based architectures may offer significant advantages in handling complex wound image classification tasks.

A key advantage of the Swin-T model lies in its ability to capture both local and global features through its hierarchical structure and sliding window self-attention mechanism (17). Unlike traditional CNNs that rely on fixed receptive fields, the Swin-T architecture can dynamically model long-range dependencies, which is particularly useful for interpreting diffuse infection characteristics such as periwound erythema, tissue necrosis, biofilm presence, or periwound inflammation. These complex visual cues are often subtle and may be difficult for non-specialist clinical personnel to accurately identify, highlighting the potential of Swin-T as a decision support tool in primary care settings.

The Swin-T model exhibited significant superiority over traditional CNN architectures (AlexNet, ResNet34, and Inception_v3) and notably outperformed non-specialist clinicians in diagnostic accuracy. Specifically, after clinical decision threshold optimization via the Youden Index, Swin-T achieved a robust balance between sensitivity (92.00%) and specificity (89.00%). This equilibrium is critical in the context of antimicrobial stewardship; while traditional models like ResNet152 or certain non-specialist clinicians may achieve higher raw sensitivity, they often do so by significantly sacrificing specificity. Such an imbalance leads to an influx of false positives, which clinically translates to the over-prescription of antibiotics and the potential exacerbation of antimicrobial resistance. By maintaining high specificity alongside strong sensitivity, the Swin-T model provides a more judicious decision-support tool, ensuring that infections are captured accurately while minimizing unnecessary medical interventions in primary care settings.

Beyond diagnostic accuracy, the operational efficiency of a model is a decisive factor for its integration into fast-paced clinical environments. Our results (Table 1) demonstrate that the Swin-T model, despite having a larger parameter capacity (86.75 M), maintains a highly competitive inference latency of 27.7 ± 2.0 ms on an NVIDIA T4 GPU. This sub-30 ms performance is significant as it supports real-time diagnostic feedback, which is essential for high-throughput screening in outpatient departments. Furthermore, the model’s stable performance on a standard CPU (486.4 ± 5.2 ms) underscores its hardware agnosticism, suggesting that the system could be reliably deployed in primary care settings or community clinics that lack dedicated GPU servers. By achieving an optimal balance between architectural depth and computational speed, this framework provides a practical solution for bridging the gap between advanced deep learning research and bedside clinical application.

However, this study has some limitations. First, this research was conducted at a single center, which may affect the immediate generalizability of the findings across diverse clinical environments. Nevertheless, this study establishes a foundational framework for broad-spectrum wound infection screening. While the dataset (*n* = 4,000) is substantial for clinical research, it is considered moderate from a deep learning perspective. To address this, we implemented data augmentation to improve model robustness. Moving forward, we have established plans for future multi-center collaborations to further optimize and validate the system across diverse geographical regions and clinical settings. Second, infection status in this study was determined based on expert consensus, and although guided by the IWII-WIC framework, some degree of subjective bias may still exist. Lastly, the models used in this study were trained and validated on static images. Future work should consider incorporating time-series images or video data to better capture dynamic wound changes and inflammation patterns.

In conclusion, this study demonstrates the feasibility and effectiveness of deep learning for automated wound infection screening using clinical images. The proposed models, including the Swin-T and conventional CNNs, achieved high diagnostic performance and reduced variability compared with non-specialist clinicians. By providing a rapid, objective, and scalable solution, these AI-driven tools have the potential to support early infection recognition, streamline clinical workflows, and improve antimicrobial stewardship. Such approaches are particularly valuable in resource-limited and decentralized healthcare settings, where they can empower non-specialist clinicians and patients, ultimately enhancing the accessibility, consistency, and quality of wound care.

## Data Availability

The data analyzed in this study is subject to the following licenses/restrictions: the dataset consists of anonymized clinical wound images collected from hospital records and is subject to patient privacy regulations. Access is restricted and cannot be publicly shared. Use of the dataset requires approval from the corresponding hospital and compliance with local ethical and legal requirements. Requests to access the dataset can be made by contacting the corresponding author, Chao Wang, at wangchaoxy@126.com. Access will require approval from the Ethics Committee of Chengdu Second People’s Hospital and compliance with all local ethical and legal requirements.

## References

[1] Uberoi A McCready-Vangi A Grice EA. The wound microbiota: microbial mechanisms of impaired wound healing and infection. Nat Rev Microbiol. (2024) 22:507–21. doi: 10.1038/s41579-024-01035-z 38575708

[2] Zhang S He W Dong J Chan YK Lai S Deng Y. Tailoring versatile nanoheterojunction-incorporated hydrogel dressing for wound bacterial biofilm infection theranostics. ACS Nano. (2025) 19:10922–42. doi: 10.1021/acsnano.4c15743, 40071724

[3] Polk HC Simpson CJ Simmons BP Alexander JW. Guidelines for prevention of surgical wound infection. Arch Surg. (1983) 118:1213–7. doi: 10.1001/archsurg.1983.01390100075019 6615204

[4] Swanson T Ousey K Haesler E Bjarnsholt T Carville K Idensohn P . IWII wound infection in clinical practice consensus document: 2022 update. J Wound Care. (2022) 31:S10–21. doi: 10.12968/jowc.2022.31.Sup12.S10, 36475844

[5] Snyder RJ Fife C Moore Z. Components and quality measures of DIME (devitalized tissue, infection/inflammation, moisture balance, and edge preparation) in wound care. Adv Skin Wound Care. (2016) 29:205–15. doi: 10.1097/01.ASW.0000482354.01988.b4, 27089149 PMC4845765

[6] Litjens G Kooi T Bejnordi BE Setio AAA Ciompi F Ghafoorian M . A survey on deep learning in medical image analysis. Med Image Anal. (2017) 42:60–88. doi: 10.1016/j.media.2017.07.005, 28778026

[7] Zhu M Pi Y Jiang Z Wu Y Bu H Bao J . Application of deep learning to identify ductal carcinoma in situ and microinvasion of the breast using ultrasound imaging. Quant Imaging Med Surg. (2022) 12:4633–46. doi: 10.21037/qims-22-46, 36060588 PMC9403599

[8] Yiğitcan Ç. Deep learning for automated breast cancer detection in ultrasound: a comparative study of four CNN architectures. AIAPP. (2025) 1:13–9. doi: 10.69882/adba.ai.2025073

[9] Alpsalaz SD Aslan E Özüpak Y Alpsalaz F Uzel H Bereznychenko V. Hybrid deep learning with attention fusion for enhanced colon cancer detection. Sci Rep. (2025) 15:45583. doi: 10.1038/s41598-025-29447-8, 41315603 PMC12754056

[10] Aslan E Demirtas Alpsalaz S Özüpak Y Alpsalaz F Uzel H. Alzheimer’s classification with a MaxViT-based deep learning model using magnetic resonance imaging. JASTT. (2025) 6, 316–27. doi: 10.38094/jastt62453

[11] Aslan E Özüpak Y. Comparison of machine learning algorithms for automatic prediction of Alzheimer disease. J Chin Med Assoc. (2025) 88:98–107. doi: 10.1097/JCMA.0000000000001188, 39965789 PMC12718816

[12] Chen H Unberath M Dreizin D. Toward automated interpretable AAST grading for blunt splenic injury. Emerg Radiol. (2023) 30:41–50. doi: 10.1007/s10140-022-02099-1, 36371579 PMC10314366

[13] Al Sadig M Alsamri J Alharbi NH Asiri MM Ghaleb M Alhashmi AA . Investigating blood cell images for enhanced hematologic disorder detection using multi-scale feature learning with a hybrid deep learning model. Sci Rep. (2025) 15:41490. doi: 10.1038/s41598-025-21412-9, 41276546 PMC12644525

[14] Yiğitcan Ç. Machine learning approaches for enhanced diagnosis of hematological disorders. CSAI. (2025) 1:8–14. doi: 10.69882/adba.csai.2025072

[15] Topol EJ. High-performance medicine: the convergence of human and artificial intelligence. Nat Med. (2019) 25:44–56. doi: 10.1038/s41591-018-0300-7, 30617339

[16] Sumithra MG Venkatesan C. SwinDFU-net: deep learning transformer network for infection identification in diabetic foot ulcer. Technol Health Care. (2025) 33:39269872:601–18. doi: 10.3233/THC-241444 39269872

[17] Chlap P Min H Vandenberg N Dowling J Holloway L Haworth A. A review of medical image data augmentation techniques for deep learning applications. J Med Imaging Radiat Oncol. (2021) 65:545–63. doi: 10.1111/1754-9485.13261 34145766

[18] Song YH Yi JY Noh Y Jang H Seo SW Na DL . On the reliability of deep learning-based classification for Alzheimer's disease: multi-cohorts, multi-vendors, multi-protocols, and head-to-head validation. Front Neurosci. (2022) 16:851871. doi: 10.3389/fnins.2022.851871 36161156 PMC9490270

[19] Alohali MA El-Rashidy N Alaklabi S Elmannai H Alharbi S Saleh H. Swin-GA-RF: genetic algorithm-based Swin transformer and random forest for enhancing cervical cancer classification. Front Oncol. (2024) 14:1392301. doi: 10.3389/fonc.2024.1392301, 39099689 PMC11294103

[20] Wang B Sun R Yang X Niu B Zhang T Zhao Y . Recognition of rare microfossils using transfer learning and deep residual networks. Biology. (2022) 12:16. doi: 10.3390/biology12010016, 36671708 PMC9854841

[21] Szegedy C Vanhoucke V Ioffe S Shlens J Wojna Z. Rethinking the inception architecture for computer vision, in Proc. IEEE Conf. Comput. Vis. Pattern Recognit. (CVPR), 2818–2826, (2016).

[22] Saboo YS Kapse S Prasanna P. Convolutional neural networks (CNNs) for pneumonia classification on pediatric chest radiographs. Cureus. (2023) 15:e44130. doi: 10.7759/cureus.44130, 37753018 PMC10518240

[23] Dosovitskiy A Beyer L Kolesnikov A Weissenborn D Zhai X Unterthiner T . An image is worth 16×16 words: transformers for image recognition at scale, [Epubh ahead of preprint]. doi: 10.48550/arXiv.2010.11929, (2020).

[24] Howard AG Zhu M Chen B Kalenichenko D Wang W Weyand T . MobileNets: efficient CNNs for mobile vision applications, [Epubh ahead of preprint]. doi: 10.48550/arXiv.1704.04861, (2017).

[25] Park SH Goo JM Jo CH. Receiver operating characteristic (ROC) curve: practical review for radiologists. Korean J Radiol. (2004) 5:11–8. doi: 10.3348/kjr.2004.5.1.11, 15064554 PMC2698108

